# Multi-omics analysis and evidence of IL1R1 as a potential biomarker for diabetes-associated intervertebral disc degeneration

**DOI:** 10.3389/fimmu.2025.1692185

**Published:** 2025-10-15

**Authors:** Meng Yi, Wancheng Lin, Shijie Liu, Yao Zhang, Jipeng Song, Yukun Yin, Lixiang Ding

**Affiliations:** ^1^ Department of Spine, Beijing Shijitan Hospital, Capital Medical University, Beijing, China; ^2^ Department of Traditional Chinese Medicine, National Cancer Center/National Clinical Research Center for Cancer/Cancer Hospital, Chinese Academy of Medical Sciences and Peking Union Medical College, Beijing, China

**Keywords:** intervetebral disc degeneration, diabetes mellitus Type 2, multiomic analyses, inflammation, scRNAseq

## Abstract

**Introduction:**

Low back pain (LBP), primarily driven by intervertebral disc degeneration (IDD), imposes a significant global health burden. While type 2 diabetes mellitus (T2DM) is a recognized risk factor for IDD, the shared molecular mechanisms remain incompletely characterized.

**Methods:**

This study employed integrated bioinformatics (WGCNA, machine learning - LASSO, RF, ANN) on human T2DM and IDD transcriptomic datasets, alongside scRNA-seq analysis of diabetic mouse nucleus pulposus (NP) tissue, to identify key drivers of diabetes-associated IDD.

**Results:**

Bioinformatics analysis of human data identified three diagnostic biomarkers (S100A12, IL1R1, FCGR2B) and constructed a robust ANN diagnostic model (AUCs: 0.744-0.868). IL1R1 emerged as the most significant risk factor. scRNA-seq revealed altered cellular composition in diabetic discs, notably increased proportion of granulocytes (predominantly neutrophils) and decreased proportion of nucleus pulposus (NP) cells. IL1R1 was highly expressed in specific diabetes-associated NP subpopulations and showed significant positive correlation with neutrophil infiltration. Functional enrichment linked IL1R1 to inflammation, DNA repair, and immune pathways. Furthermore, we constructed a regulatory network (STAT1/STAT6-IL1R1-miRNAs-lncRNAs) and identified icariin as a potential therapeutic candidate via molecular docking.

**Discussion:**

These findings establish IL1R1 as a pivotal molecular bridge connecting T2DM and IDD, driven by neutrophil-mediated inflammation and NP cell dysfunction, offering novel diagnostic and therapeutic avenues.

## Introduction

Low back pain, primarily caused by intervertebral disc degeneration (IDD), imposes a substantial economic burden exceeding $100 billion annually in direct and indirect costs (1–3). It not only severely impairs patients’ quality of life but also places significant pressure on society. The multifactorial etiology of IDD complicates the development of personalized prevention and treatment strategies (4–6). Among these factors, metabolic disturbances within the intervertebral disc are a major contributor to degeneration (7, 8).

As the most prevalent systemic metabolic disorder, type 2 diabetes mellitus (T2DM) significantly threatens elderly populations (9). While diabetes complications such as retinopathy and nephropathy are well-established, emerging evidence implicates diabetes-induced metabolic dysregulation and systemic inflammation in IDD pathogenesis (10–12). Elevated glucose levels stimulate advanced glycation end product (AGE) accumulation, and excessive AGEs mediate dysregulated proteoglycan synthesis and disc fibrosis (13). Additionally, diabetic microangiopathy may compromise nutrient delivery to disc tissues, reducing nutrient availability and impairing metabolic waste clearance (14). Nevertheless, investigations into shared genetic and molecular mechanisms linking diabetes and IDD remain limited.

To address this gap, this study employs bioinformatics approaches to identify common biomarkers associated with both T2DM and IDD. By integrating weighted gene co-expression network analysis (WGCNA), machine learning, single-cell sequencing (scRNA-seq) analysis, and protein docking, we aim to determine key genes involved in this process, thereby providing novel insights into the pathogenesis of diabetes-associated intervertebral disc degeneration.

## Materials and methods

### Public data sources

All data were obtained from the Gene Expression Omnibus (GEO) database (https://www.ncbi.nlm.nih.gov/geo/) (**Supplementary Table 1**). The dataset GSE7014 comprised microarray data from 36 patients, from which 10 T1DM samples were excluded, retaining 26 samples (20 T2DM and 6 normal samples) for subsequent analysis. For IDD, expression data were integrated from two independent datasets, GSE124272 and GSE34095, resulting in a combined set of 11 degenerated disc samples and 11 normal controls.

### Batch effect correction and quality control

To mitigate technical variations between datasets, we applied a systematic normalization and batch correction pipeline. Expression data were merged by gene symbol and normalized using the quantile method via the limma package. Batch effects were subsequently corrected using the removeBatchEffect function from limma. Correction efficacy was evaluated using principal component analysis (PCA) and expression distribution boxplots. PCA was carried out on scaled data using prcomp, and results were visualized through scatter plots with batch-wise confidence ellipses.

**Figure 1:**
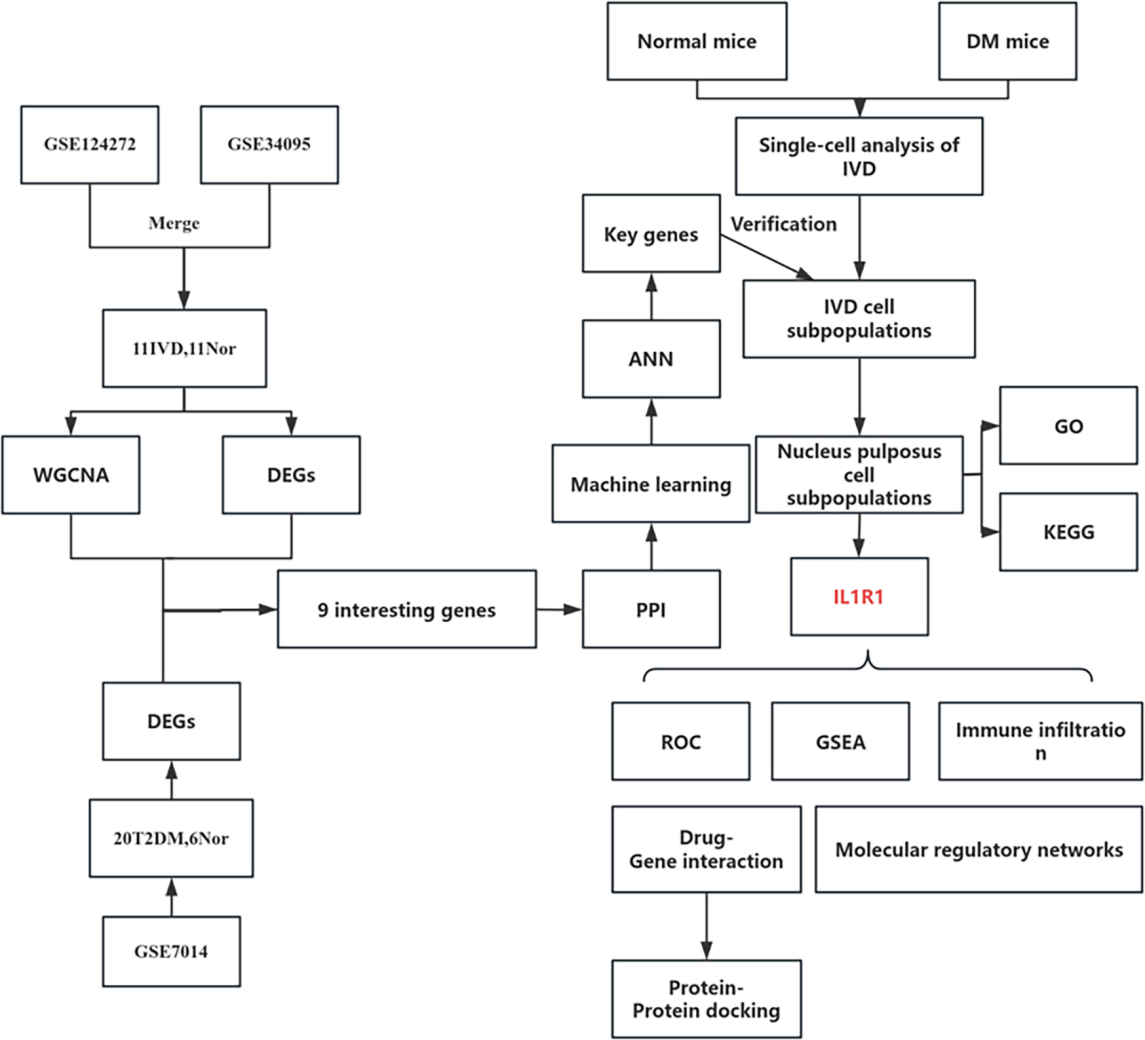
Flowchart of the study.

### Diabetes mouse model and scRNA-seq processing

All animal experiments were approved by the Ethics Committee of Beijing Shijitan Hospital, Capital Medical University (Approval No. Sjtkyll-lx-2021105), and were conducted in accordance with the institutional guidelines for the care and use of laboratory animals. ScRNA-seq data were generated from nucleus pulposus cells of 10 C57BL/6 mice. Diabetes was first induced in all mice by a high-fat/high-sucrose diet and a single intraperitoneal injection of streptozotocin (55 mg/kg). Thereafter, mice were divided into two groups (5 vs. 5): One group remained diabetic, whereas the other received treatment to achieve normoglycemia and served as the treated control group. NPCs were isolated following the experimental protocol described by Andrew Bratsman et al. (15).

Briefly, nucleus pulposus tissues were minced and washed with RPMI-1640 medium (Gibco, C11875500137). Tissue dissociation was performed using a vascular tissue dissociation kit (Bestopcell, BA3310) according to the manufacturer’s instructions. After digestion, the cell suspension was filtered through a 70-μm nylon strainer (Thermo Fisher Scientific, 22-363-548) to remove debris and centrifuged at 300 × g for 5 min at 4 °C. The pellet was resuspended in PBS (Bioss, C7033) supplemented with 5% fetal bovine serum (FBS; Solarbio, s9020). Red blood cells were lysed using a red blood cell lysis buffer, followed by incubation for 15 min at 4 °C and centrifugation at 450 × g for 5 min at 4 °C. The cell pellet was washed twice with PBS containing 5% FBS and resuspended in the same buffer. Cell concentration and viability were assessed using CountStar, and the suspension was adjusted to a density of 600–1,200 living cells per microliter.

Single-cell encapsulation and library preparation were performed using the CelCode^®^ Single Cell 3′ Transcriptome Kit v1.0 following the manufacturer’s protocol. Approximately 70 μL of master mix and cell suspension, 50 μL of barcoded gel beads, and 45 μL of partitioning oil were loaded into the CelCode^®^ Driver to generate gel bead-in-emulsions (GEMs). Reverse transcription was performed to synthesize barcoded cDNA, which was then amplified by PCR. Sequencing libraries were constructed and normalized before being sequenced on an MGI DNBSEQ-T7 platform with paired-end 150-bp reads (PE150), achieving a minimum depth of 50,000 reads per cell. All sequencing services were provided by Bestopcell (Beijing).

Raw sequencing data were demultiplexed and aligned to the reference genome using the CelScene^®^ pipeline with default parameters. The feature-barcode matrix was generated using the CelScene^®^ count module, which performed alignment, filtering, barcode assignment, and UMI counting. Dimensionality reduction was conducted via PCA, and the top 10 principal components were used for cluster identification using both K-means and graph-based clustering algorithms.

### Differential gene screening

Differentially expressed genes (DEGs) in T2DM and IDD were identified using the limma package in R, with thresholds set at |log_2_ fold change (FC)| > 0.5 and an adjusted p-value < 0.05. Volcano plots and heatmaps were generated using the ggplot2 and pheatmap packages.

### WGCNA

WGCNA was employed to identify clinically relevant co-expression gene modules (16). First, gene expression data were preprocessed by filtering highly variable genes based on median absolute deviation (MAD > 0.5), and outlier samples were removed via hierarchical clustering. The pickSoftThreshold function in the WGCNA package was used to determine the optimal soft-thresholding power (β) for constructing a scale-free topology network. A weighted adjacency matrix was then computed and converted into a topological overlap matrix (TOM) to assess gene co-expression connectivity.

Hierarchical clustering based on TOM dissimilarity was performed to group genes with similar expression patterns into modules. Dynamic tree cutting (height = 0.25) was applied to merge closely related modules. Module eigengenes (MEs) were calculated, and their correlations with clinical traits were analyzed to identify key modules associated with disease phenotypes.

### PPI network analysis

The STRING database was utilized to construct protein–protein interaction (PPI) networks, visualized using Cytoscape (v3.10.3). Module analysis was performed with the MCODE plugin, and hub genes were identified using the cytoHubba plugin.

### Machine learning

Advanced machine learning algorithms were employed to develop a predictive model for diabetes-associated IDD. Least absolute shrinkage and selection operator (LASSO) regression was implemented using the glmnet package (17). The Random Forest (RF) algorithm was executed via the randomForest package in R (18). Additionally, an artificial neural network (ANN) model was constructed using the neuralnet and neuralnettools packages (19).

### Immune infiltration

The relative abundance of 22 immune cell subtypes in these samples was estimated using the CIBERSORT algorithm (20). Pearson correlation analysis was conducted to examine associations between key genes and immune cell subtypes.

### Molecular regulatory network of key genes

The HTFtarget database predicted transcription factors interacting with key genes (21). mRNA–miRNA interactions were explored using PITA, microT, and TargetScan, with consensus predictions retained. MiRNet, Starbase, and LncBase v3 identified miRNA-lncRNA interactions, validated if consistent across databases (22–24). LncACTdb 3.0 predicted lncRNA-transcription factor interactions (25).

### Drug–gene interactions and protein–protein docking

DrugBank analyzed drug–gene interactions, whereas PyMOL facilitated protein–protein docking and molecular visualization (26).

### Statistical analysis

All data processing and analyses were performed using R software (Version 4.4.1). For bulk RNA-seq differential expression analysis, the limma package was used. For comparisons between two groups in other analyses, the Wilcoxon test was applied. Differences in IL1R1 expression across NP cell subclusters were assessed using the Kruskal–Wallis test. Spearman correlation analysis was conducted to examine the relationship between key genes and immune cells, with a p-value < 0.05 considered statistically significant.

## Results

### Identification of DEGs in IDD and T2DM

**Figure 2:**
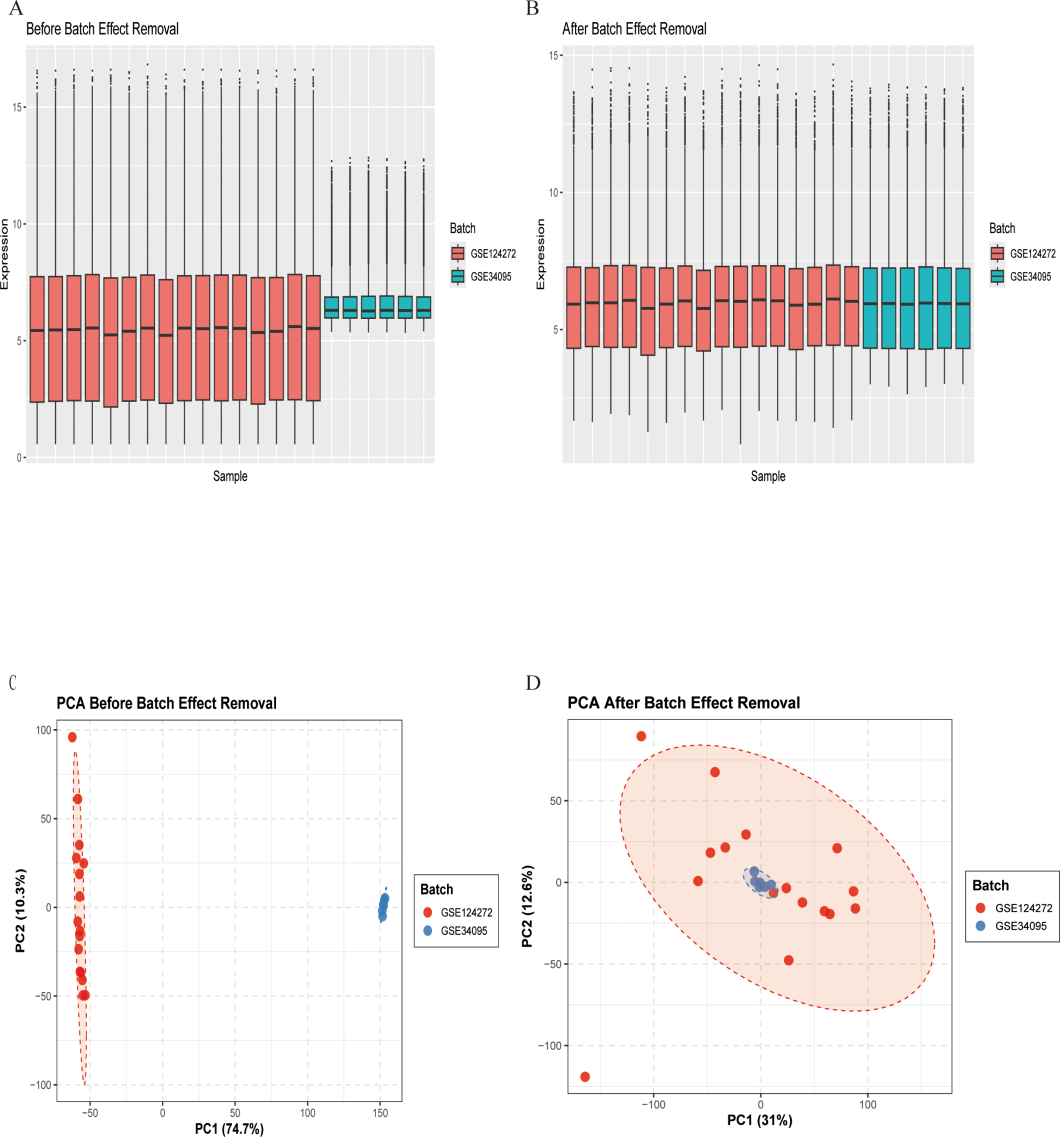
Gene expression data before and after batch effect removal. **(A)** Box plot before batch effect removal. **(B)** Box plot after batch effect removal. **(C)** PCA results before batch effect removal. **(D)** PCA results after batch effect removal.

**Figure 3:**
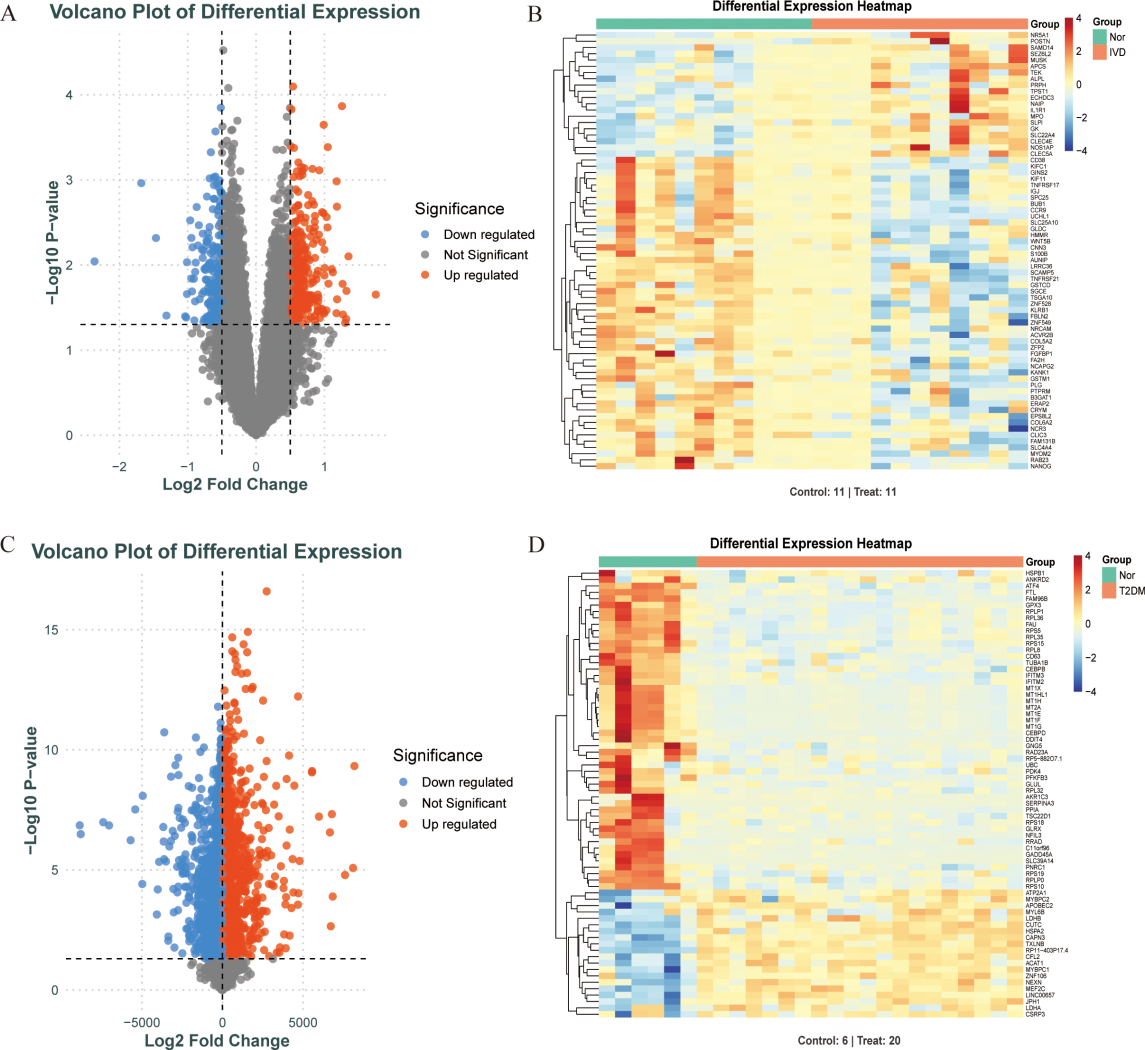
Screening DEGs of diabetes and IDD. **(A, B)**. When comparing IDD (n=11) and normal (n=11) samples, a volcano plot and heatmap for DEGs is shown. **(C, D)**. When comparing T2DM (n=20) and normal (n=6) samples, a volcano plot and heatmap for DEGs is shown.

**Figure 1** depicts the study flowchart. The expression profile dataset GSE7014 was normalized. For the IDD datasets (GSE124272 and GSE34095), raw data were integrated, and batch effects were corrected using the removeBatchEffect function from the limma R package. The efficacy of this batch effect correction was assessed using PCA, as visualized in **Figure 2**. After merging the batch-corrected data, normalization was performed, followed by the generation of volcano plots and heatmaps for differential expression analysis of two datasets (**Figure 3**). In the figure, red dots represent significantly upregulated differentially expressed genes, and blue dots represent significantly downregulated genes.

Construction of WGCNA and gene module screening

**Figure 4:**
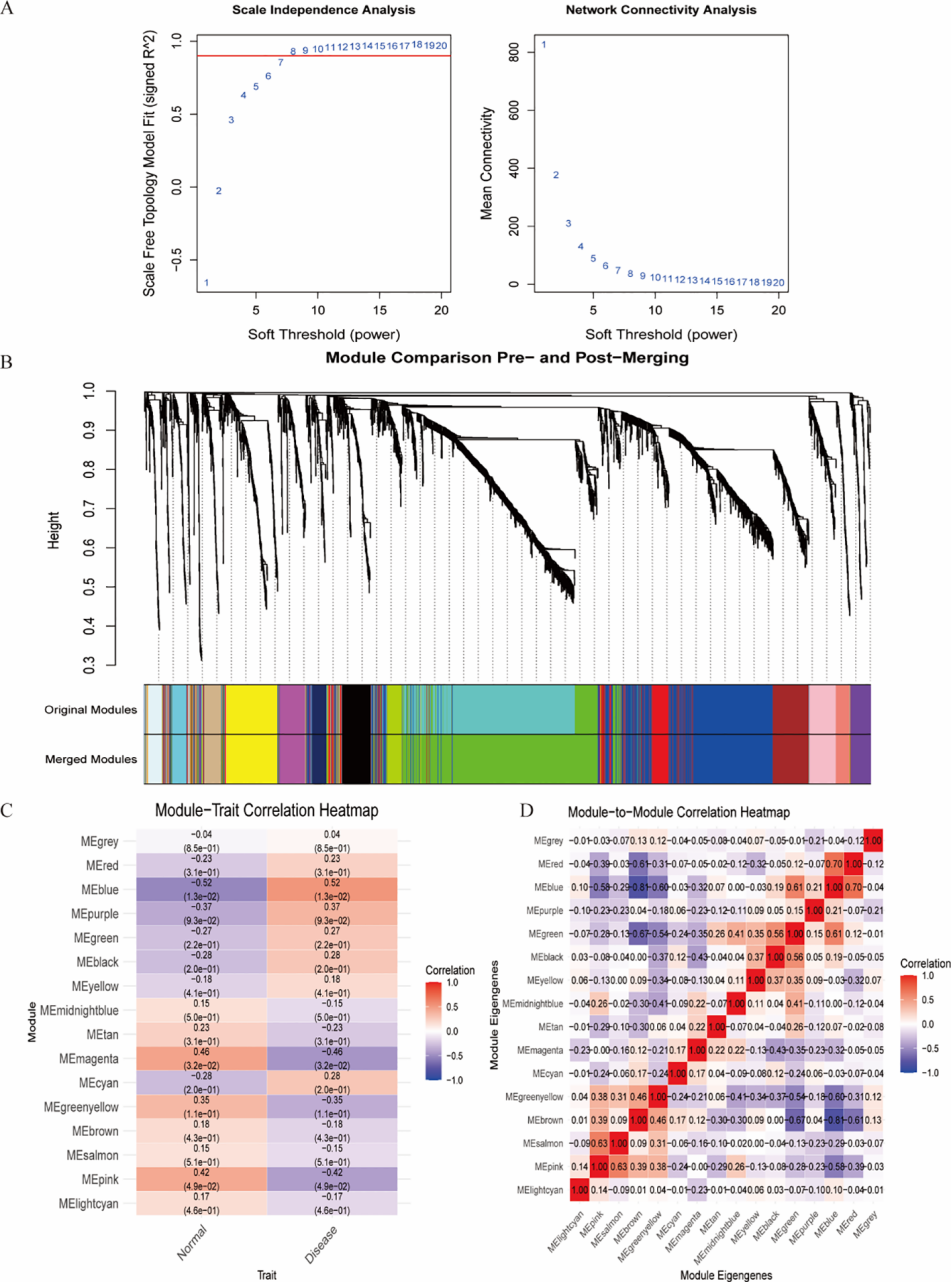
Identification of the most associated module genes via WGCNA in IDD. **(A)** Based on the results of scale independence and average connectivity, β=8 was selected as the best soft threshold. **(B)** Gene co-expression modules are displayed in various colors beneath the gene tree. **(C)** Heatmap of the association between modules and IDD. **(D)** Heatmap of the association between different modules.

The WGCNA algorithm was utilized to identify key gene modules closely associated with IDD, with the soft thresholding power (β) set to 8. The dynamic tree-cutting algorithm delineated 16 gene modules, among which the blue module exhibited the most significant correlation (correlation coefficient = 0.56) compared with other modules. Consequently, the blue module was selected as the key module for subsequent analyses (**Figure 4**).

### Protein–protein interaction network construction

**Figure 5:**
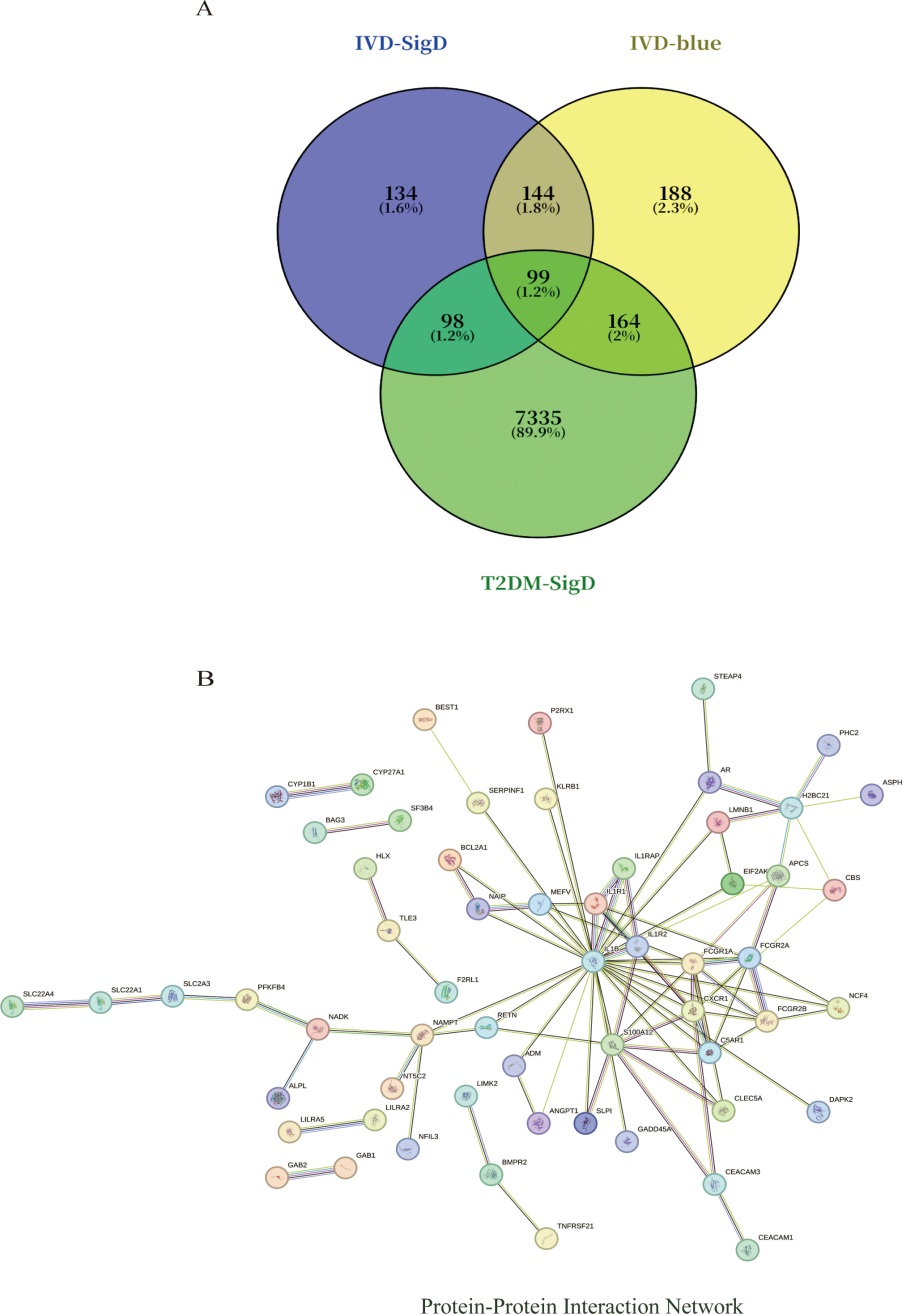
**(A)** Venn diagram shows that there are 99 intersected genes among DEGs of IVD, blue module genes of IVD, and DEGs of T2DM. **(B)** The relationship among these genes in the PPI network.

**Figure 6:**
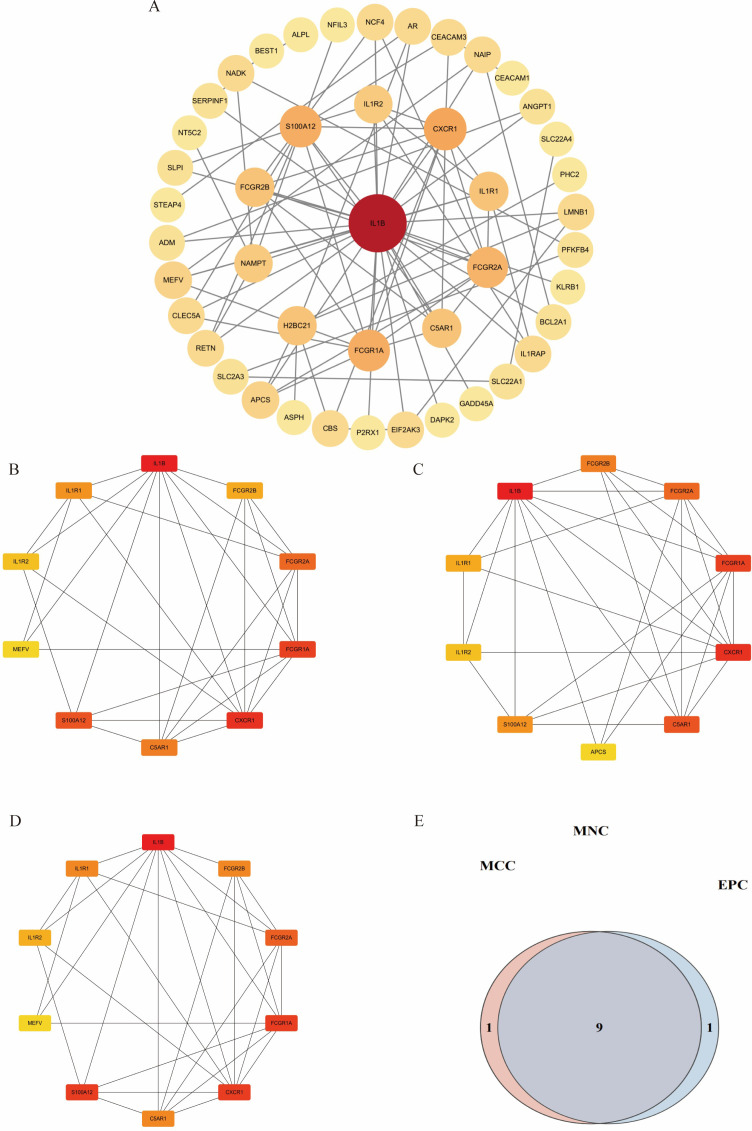
Visualization of different algorithms in PPI network. **(A)**. The first module of the PPI network. **(B–D)**. Visualization of the MNC, MCC, and EPC algorithms. **(E)**. Nine common hub genes were identified by different algorithms of cytoHubba plugin.

Intersection analysis of DEGs related to IDD, genes from the IDD-associated blue module, and T2DM-related DEGs revealed nine overlapping genes. Given that genes and their encoded proteins may interact, a PPI network was constructed using the STRING tool, comprising 99 nodes (**Figure 5**). To identify the most interconnected module within the network, the MCODE plugin was applied, yielding 45 key nodes. Further analysis using the cytoHubba plugin with MCC, MCN, and EPC algorithms identified nine common hub genes: IL1B, CXCR1, FCGR1A, S100A12, FCGR2A, C5AR1, IL1R1, FCGR2B, and IL1R2 (**Figure 6**).

### Identification and validation of key genes in diabetes-associated IDD

**Figure 7:**
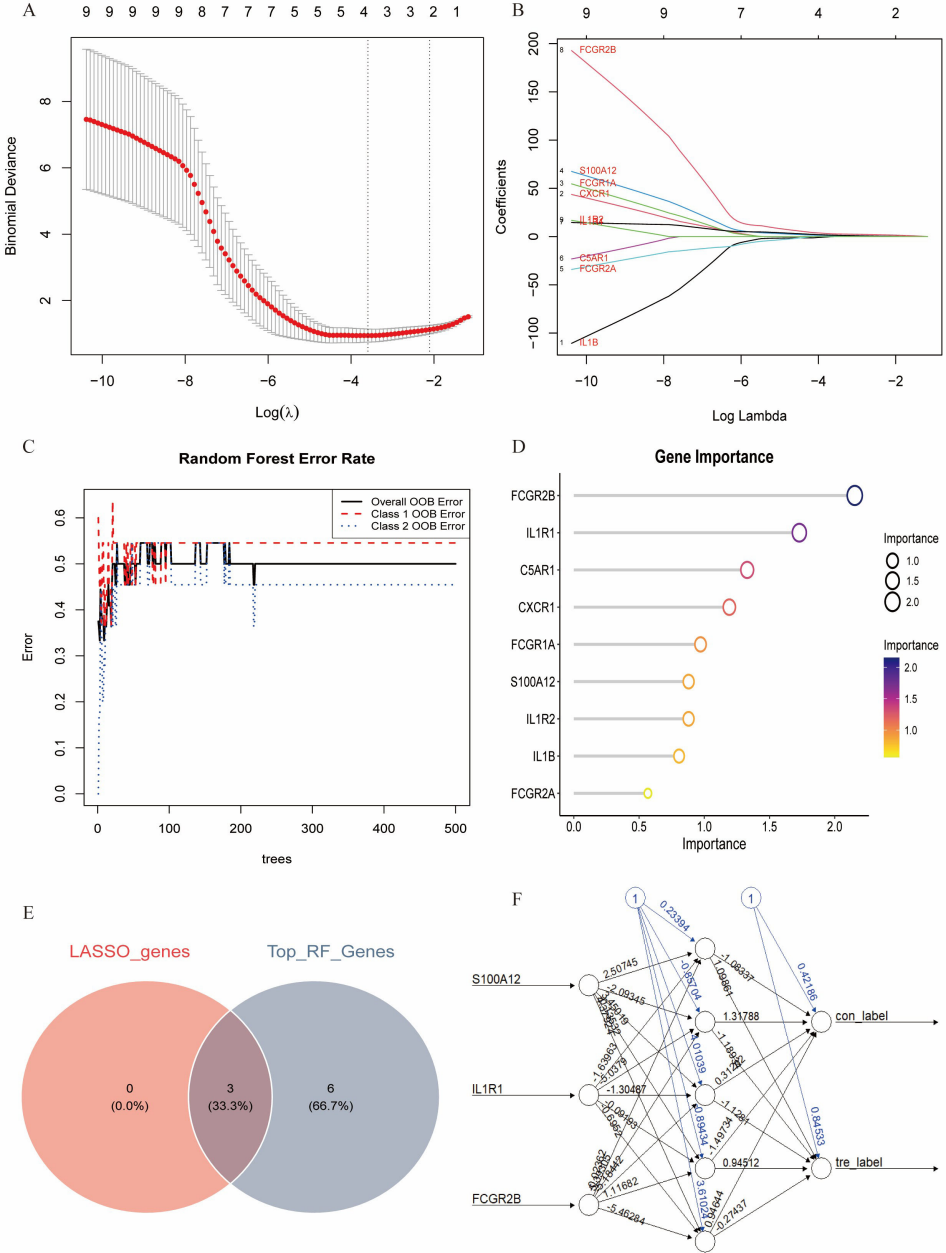
Identification of the key genes. **(A, B)**. Key genes were identified form hub genes by machine learning LASSO regression. **(C, D)**. Key genes were identified from hub genes by the Random Forest (RF) algorithm. **(E)**. S100A12, IL1R1, and FCGR2B were identified by overlapping. **(F)**. Artificial neural networks (ANN) model of the key genes.

**Figure 8:**
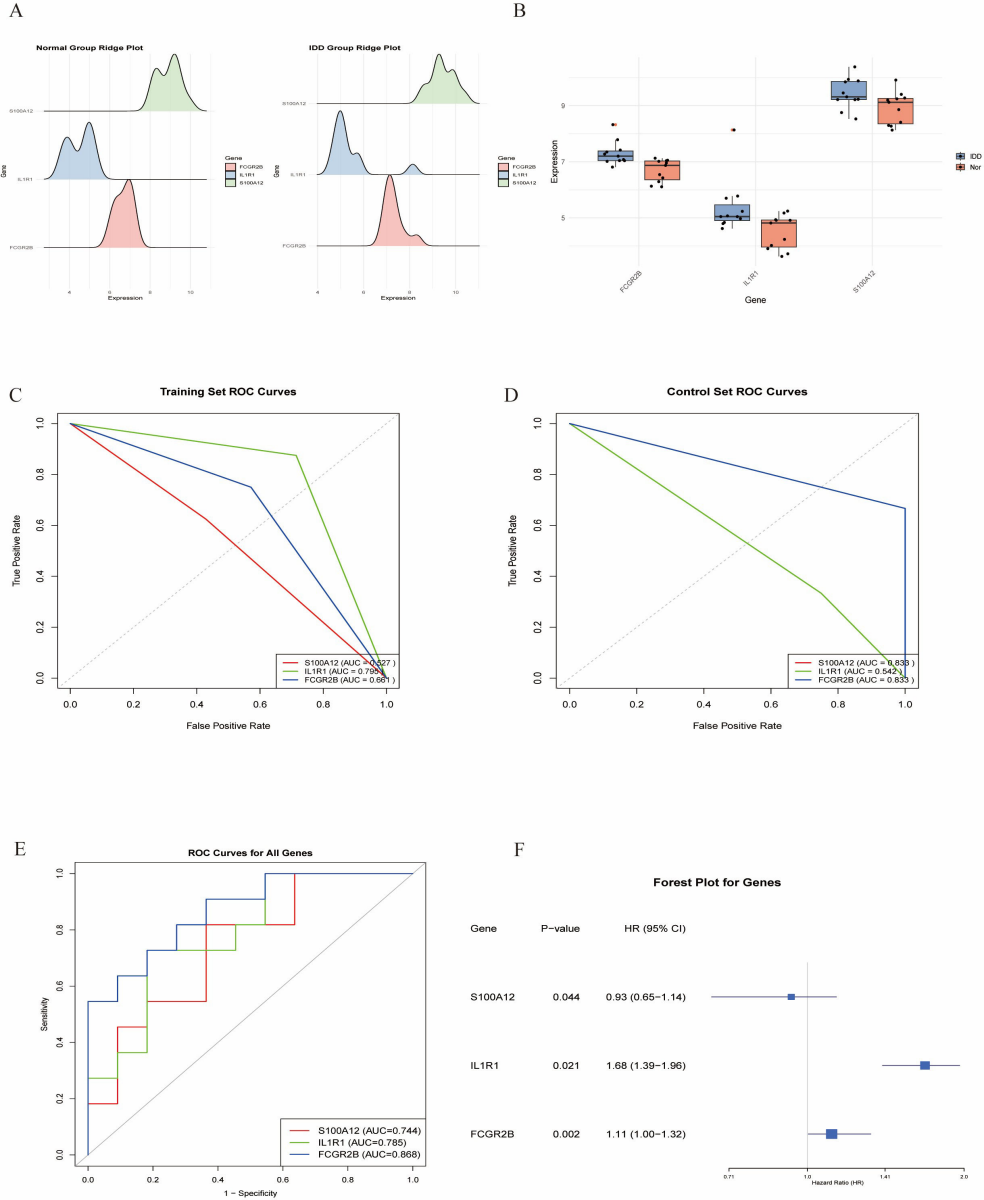
Validation of the key genes. **(A,B)**. The key genes are significantly expressed in IDD. **(C, D)**. ROC curves of the key in the training set and control set. **(E)**. ROC analysis of the key genes of ANN model. **(F)**. Forest plot of the key genes in IDD.

Multiple machine learning algorithms were employed to screen reliable biomarkers for diabetes-associated IDD. The LASSO regression algorithm identified three potential diagnostic markers, whereas the RF algorithm detected nine diagnostic genes. Venn diagram analysis ultimately revealed three overlapping diagnostic markers: S100A12, IL1R1, and FCGR2B. The ANN diagnostic model was established based on gene weights, and its performance was evaluated using receiver operating characteristic (ROC) curves (**Figure 7**). The area under the curve (AUC) values for S100A12, IL1R1, and FCGR2B were 0.744, 0.785, and 0.868, respectively, indicating robust diagnostic efficacy for diabetes-associated IDD. Although all three genes exhibited statistically significant differences between the normal and IDD groups, forest plot analysis demonstrated that IL1R1 was the most prominent risk factor specific to IDD (**Figure 8**).

### scRNA-seq analysis

**Figure 9:**
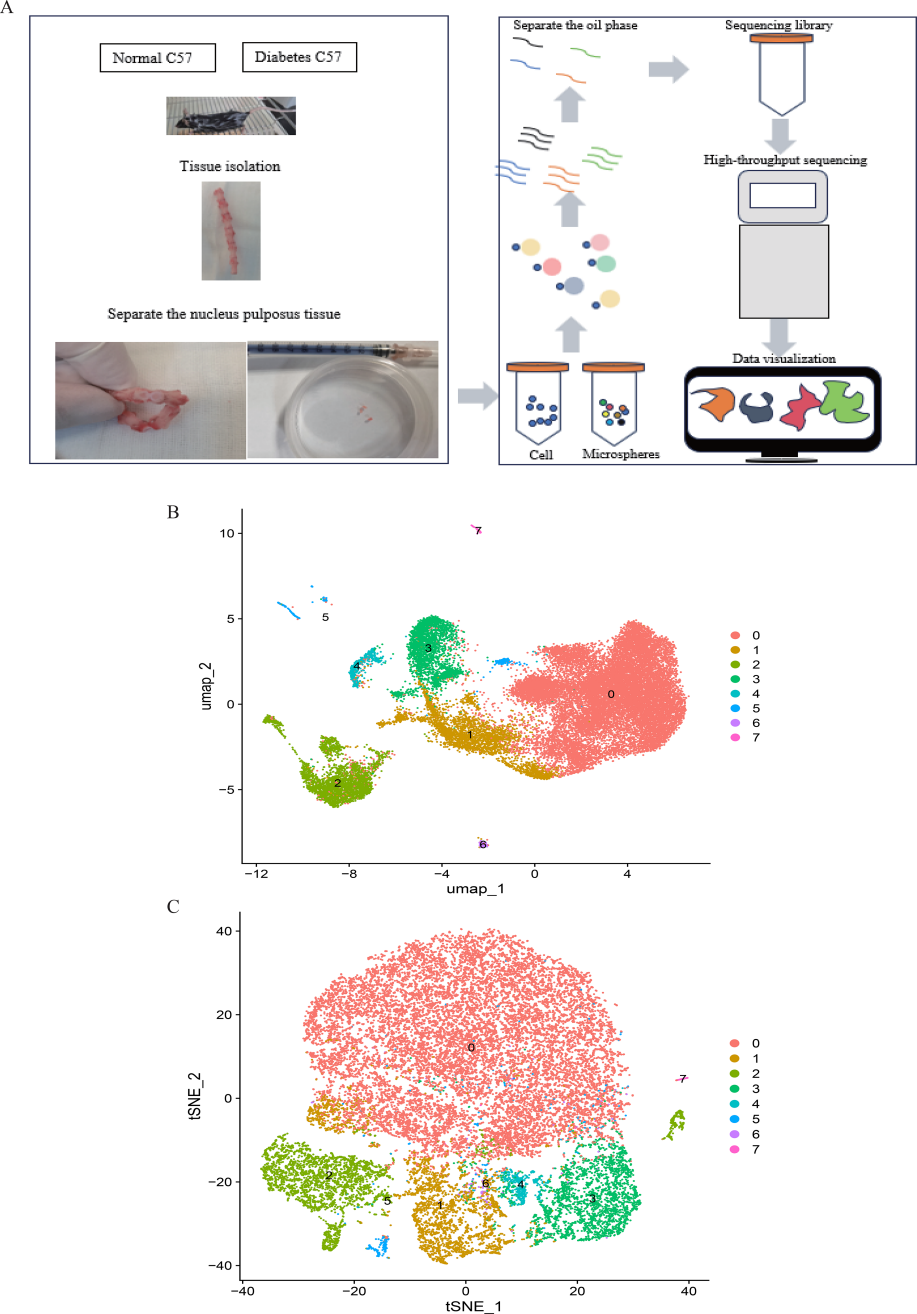
**(A)**. Single-cell RNA sequencing of nucleus pulposus cells extracted from 10 mice reveals the heterogeneity of disc cells. **(B, C)**. Umap and tSNE plot showing the unbiased classification.

**Figure 10:**
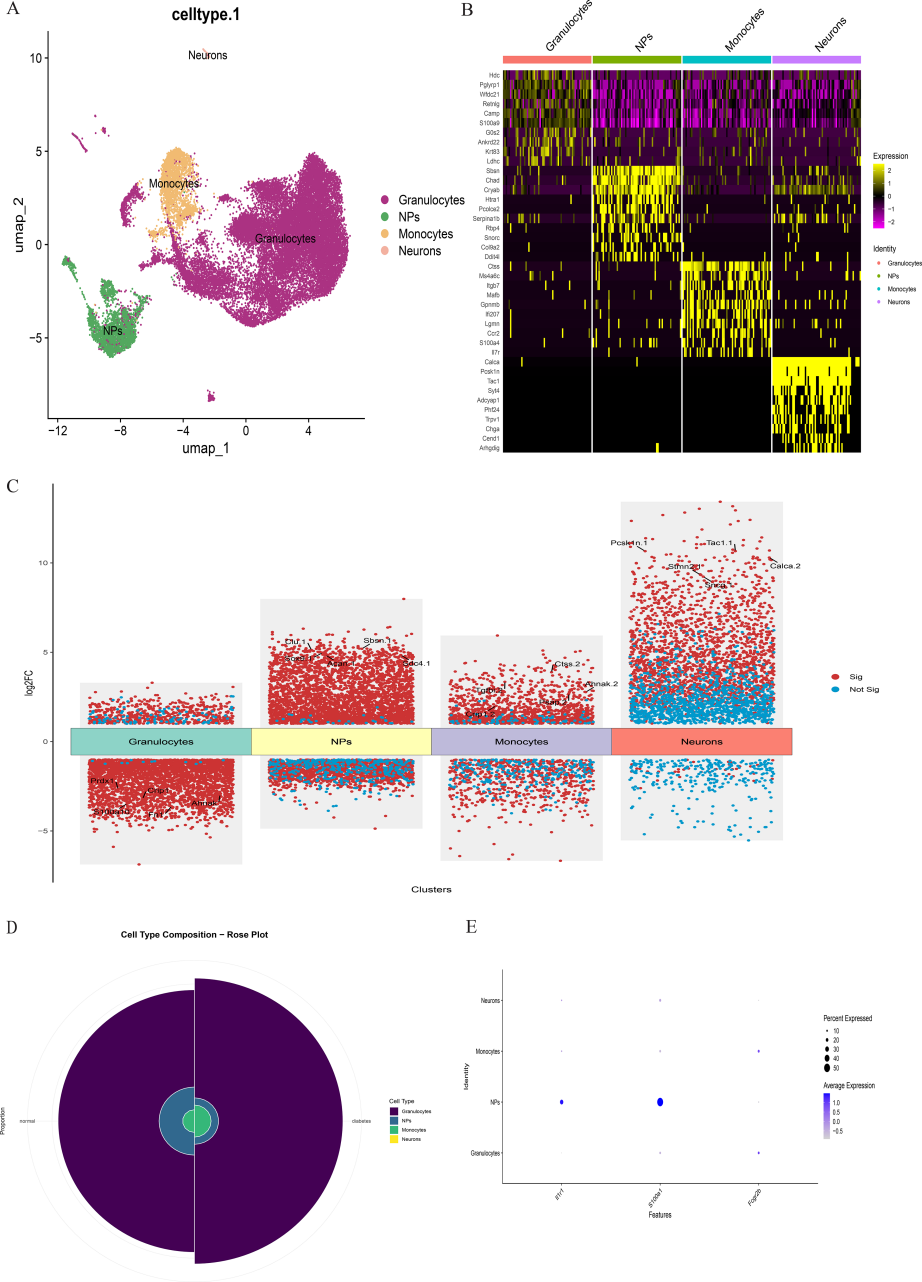
**(A)** The intervertebral disc cells were clustered into four groups using UMAP based on the expression of specific genes. **(B)** Heatmap showing differentially expressed genes in four clusters. **(C)** Differentially expressed gene heatmap. **(D)** Rose plot indicating the heterogeneity of cell cluster percentage between normal sample and diabetes sample. **(E)** Key gene expression in subclusters.

scRNA-seq data underwent standardized processing, including quality control, normalization, and unsupervised dimensionality reduction clustering. Principal component analysis and a resolution of 0.1 yielded eight cell clusters (**Figure 9**). Based on cluster-specific markers, four major cell types were identified in diabetic mouse intervertebral discs: granulocytes (most abundant, highly expressing S100A10, FN1, PRDX1, CRIP1, and AHNK), NP cells (highly expressing ACAN, COL1A1, CLU, SOX9, SBSN, and SDC4), monocytes (highly expressing PSAP, CTSS, and TGFBI), and neurons (highly expressing PCSK1N, STMN2, SNCG, CALCA, and TAC) (**Figure 10**).

**Figure 11:**
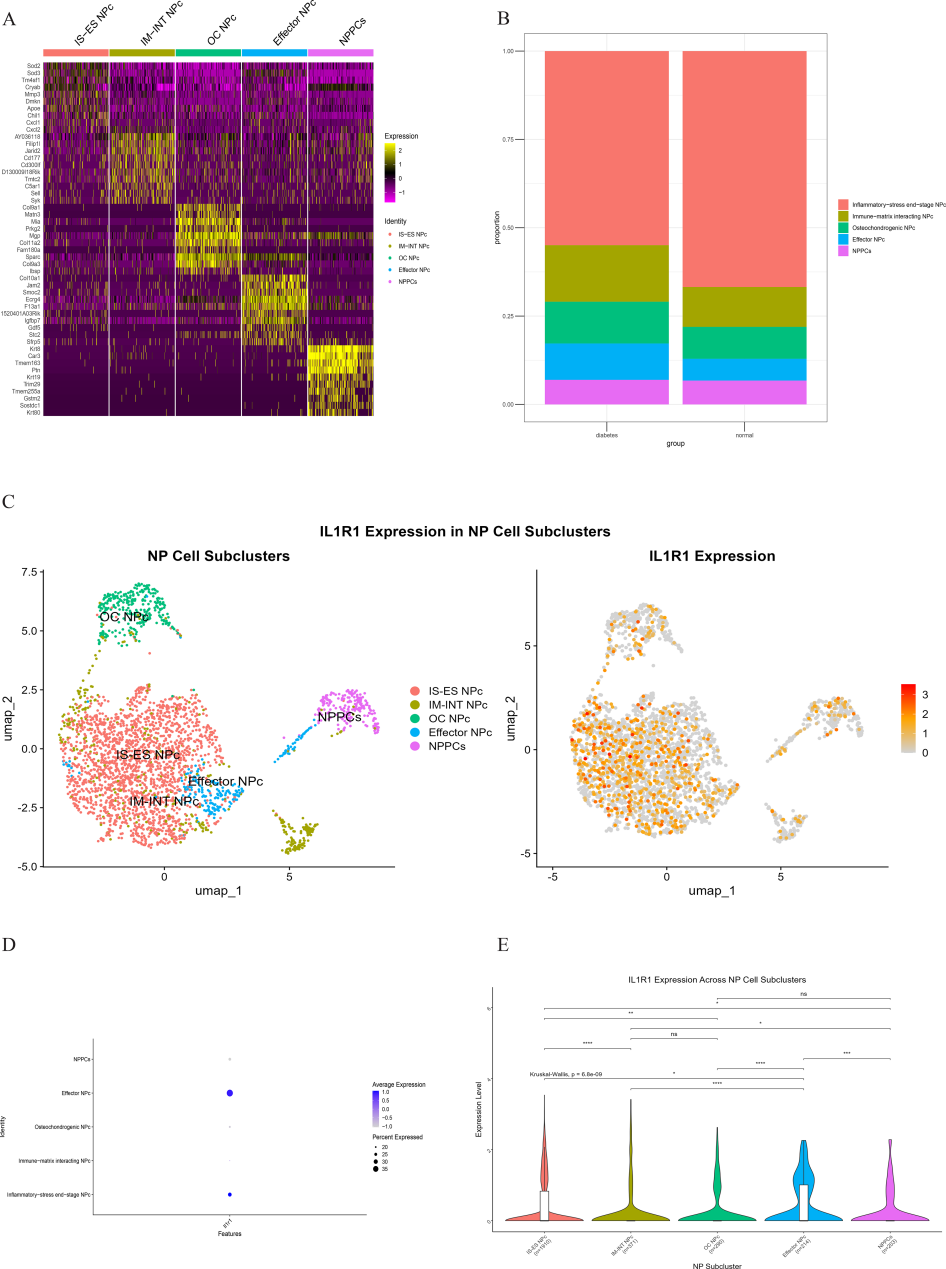
**(A)** Heatmap showing differentially expressed genes in five clusters of the NP cell. **(B)** Heterogeneity of cell cluster percentage in different samples. **(C)** UMP plot displays the characteristics of subcluster cells and the expression of core gene IL1R1. **(D)** Key gene IL1R1 expression in various subclusters. **(E)** Differential expression of the core gene IL1R1 in different subclusters.

**Figure 12:**
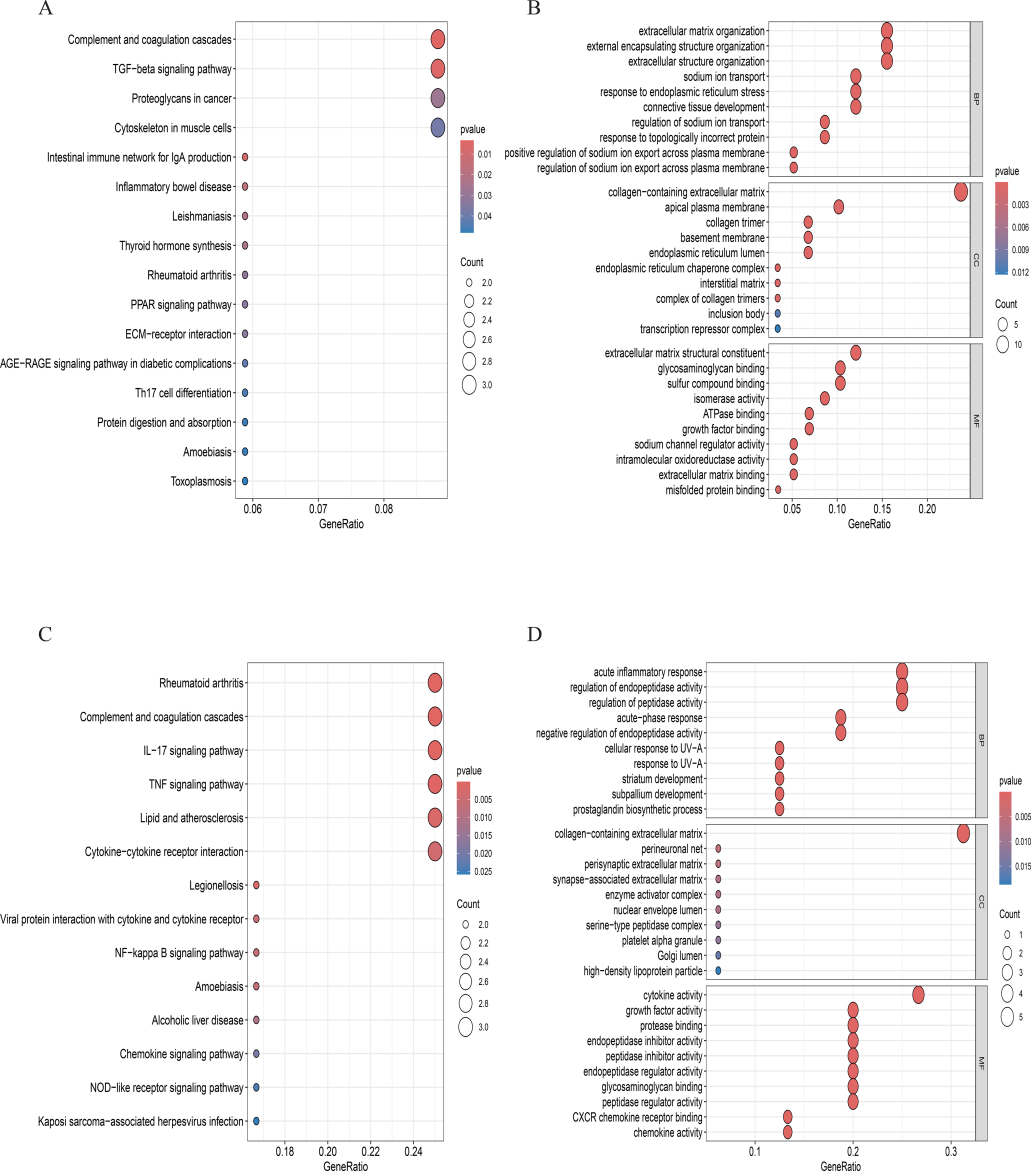
**(A, B)**. GO and KEGG enrichment analyses in effector NP cells. **(C, D)**. GO and KEGG enrichment analyses in inflammatory stress-related NP.

Comparative analysis of overall cell proportions revealed that diabetic mice exhibited a significant increase in granulocytes and monocytes and a decrease in NP cells compared with controls. Further analysis of the core gene IL1R1 demonstrated its high expression in NP cells. Subsequent subclustering of NP cells identified five distinct subpopulations based on highly expressed genes: NP progenitor cells (NPPCs, highly expressing KRT8, KRT18, KRT19, and DSP), effector NP cells (effector NPc, highly expressing FGF21, STC2, NPHS2, and WNT7B), immune-matrix interacting NP cells (IM-INT NPc, expressing S100A8/A9, ADAM8, and THBS1), inflammatory-stress end-stage NP cells (IS-ES NPc, highly expressing CXCL1, CXCL2, SAA1, SAA2, and GAS6)), and osteochondrogenic NP cells (OC NPc, highly expressing SP7, ALPL, IBSP, and VIT). Analysis revealed an expansion of effector NP and IS-ES NP subpopulations in diabetic conditions (**Supplementary Table 2**). Within the NP compartment, these two subpopulations served as the predominant hubs of IL1R1 expression, with levels significantly exceeding those in other NP subtypes (**Figure 11**). KEGG and GO enrichment analyses of these subpopulations revealed their involvement in pathways such as the complement and coagulation cascade, TGF-β signaling, and rheumatoid arthritis, as well as biological processes including extracellular matrix organization and acute inflammatory response (27) (**Figure 12**).

### Immune infiltration, molecular regulatory network, and drug interaction analysis of IL1R1

The CIBERSORT algorithm was applied to investigate immune cell infiltration in diabetes-associated IDD samples across 22 immune cell types. Compared with controls, IDD samples exhibited statistically significant differences in plasma cells and activated NK cells. Further analysis of the core gene IL1R1 and its relationship with immune cell infiltration revealed a positive correlation with neutrophils and negative correlations with activated NK cells and dendritic cells.

**Figure 13:**
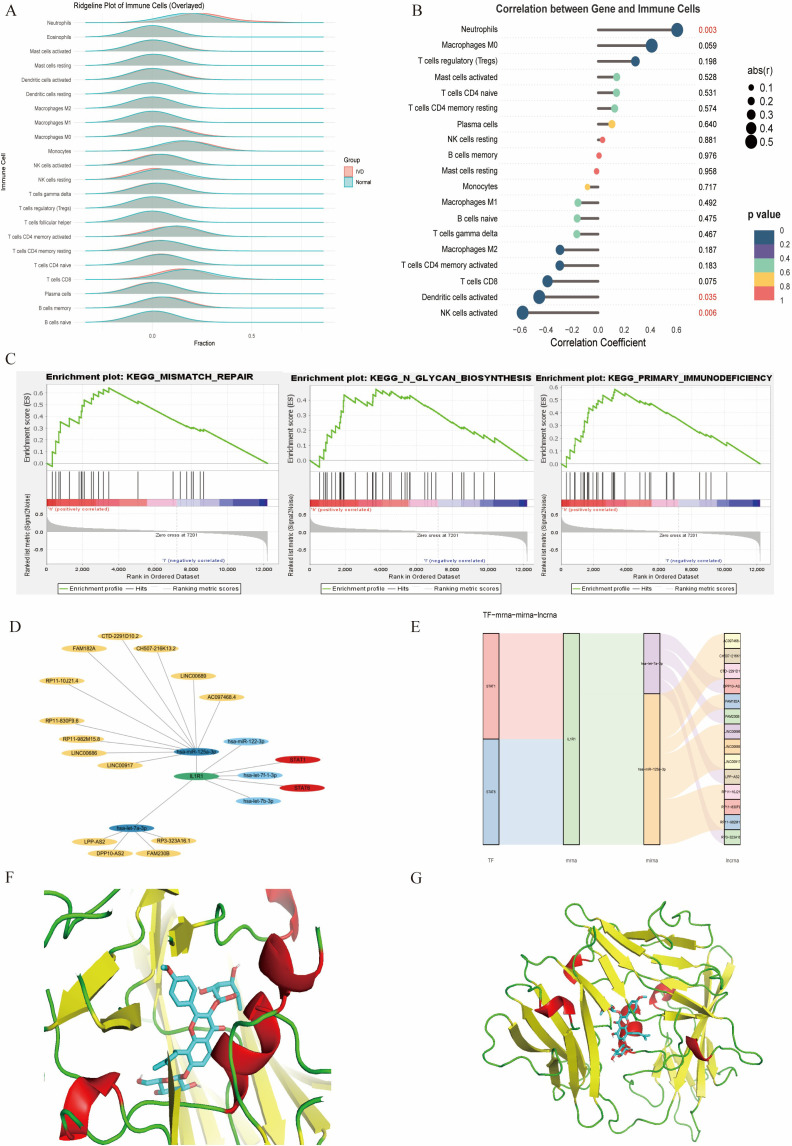
**(A)** Immune infiltration analysis. **(B)** Association between IL1R1 and different immune cells. **(C)** GSEA of IL1R1. **(D)** Circular regulatory signaling pathway. **(E)** Alluvial diagram of the TF–mRNA–miRNA–lncRNA network. **(F, G)**. Icariin–IL1R1 interaction analysis. Protein–protein docking between IL1R1 and targeting drug (blue).

To elucidate the characteristics of diabetes-associated IDD, gene set enrichment analysis (GSEA) of IL1R1 was performed, highlighting its association with seven key pathways: DNA mismatch repair, N-glycan biosynthesis, primary immunodeficiency, base excision repair, DNA replication, homologous recombination, and asthma. A regulatory network encompassing TFs/mRNAs/miRNAs/lncRNAs was constructed, including axes such as STAT1/IL1R1/hsa-let-7a-3p/lncRNA and STAT2/IL1R1/hsa-miR-125a-3p/lncRNA. The cytoHubba plugin identified lncRNA-involved regulatory circuits. DrugBank database screening nominated icariin as a candidate therapeutic compound. Additionally, the binding pocket of IL1R1 with potential drugs was predicted (**Figure 13**).

## Discussion

The etiology of IDD is multifactorial, involving both endogenous genetic susceptibility and exogenous stress factors such as aging, mechanical overload, nutritional deficiency, and notably, metabolic disorders (14). Diabetes mellitus, as a systemic metabolic disease, directly or indirectly contributes to alterations in the metabolic environment of various organs (10). Multiple clinical studies have demonstrated a strong positive correlation between T2DM and IDD, with longer disease duration and poorer glycemic control associated with more severe disc degeneration (11). Fundamental research suggests that elevated glucose levels lead to increased accumulation of AGEs, which induce structural changes in the cartilage endplate, ultimately resulting in nutrient deprivation of NP cells and an elevated risk of IDD (13). Although an association between these two conditions has been established, studies utilizing bioinformatics and machine learning to identify diagnostic biomarkers linking IDD and diabetes remain limited.

In this study, we analyzed gene expression profiles associated with T2DM and IDD to identify shared pathogenic genes. WGCNA was employed to discern gene modules specific to IDD, facilitating further screening of IDD-related gene expression. Through intersection analysis of module genes and DEGs, nine hub genes were precisely identified using multiple algorithms. Machine learning methods subsequently pinpointed three key genes: IL1R1, S100A12, and FCGR2B. Based on these genes, an artificial neural network diagnostic model was developed, which exhibited robust predictive performance even in models of non-diabetic disc degeneration. In clinical cohorts, it is challenging to isolate the effect of diabetes on IDD progression due to the frequent presence of confounding comorbidities in diabetic patients. Moreover, the systemic complications of diabetes often manifest over prolonged periods, posing challenges in translating high-quality clinical findings into targeted interventions for diabetes-associated IDD. Our approach offers distinct advantages by leveraging multi-omics analysis and machine learning to identify key protein factors at the genetic level, thereby laying the groundwork for potential therapeutic targets. Notably, these key genes were identified for the first time in the context of diabetes-associated IDD, and ROC analysis confirmed their diagnostic potential.

To investigate the direct and indirect effects of diabetes on intervertebral discs, we established a T2DM mouse model and performed scRNA-seq on NP tissues. The sequencing results show four distinct cell subpopulations: NP cells, granulocytes, neurons, and monocytes. The proportion of NP cells was significantly higher in diabetic treatment control mice, whereas granulocytes and monocytes were more abundant in diabetic mice. Further examination of key gene expression across cell types demonstrated marked differential expressions of IL1R1 and S100A12, with IL1R1 upregulated in diabetic mice and S100A12 elevated in controls. Given the central role of NP cells in disc function and degeneration, we prioritized IL1R1 for subsequent investigation. Subclustering of NP cells identified five subpopulations: NPPCs, effector NP cells, IM-INT NP cells, IS-ES NP cells, and OC NP cells. Diabetic mice exhibited altered proportions of all subpopulations except NPPCs, with increased frequencies of effector NP cells, IM-INT NP cells, and OC NP cells. IL1R1 was highly expressed in effector and IS-ES NP cells. Mechanistically, IL1R1 binds interleukin-1 (IL-1) and recruits IL-1 receptor accessory protein (IL-1RAP), initiating a signaling cascade via Toll/interleukin-1 receptor (TIR) domains that activates MyD88 and IRAK, thereby mediating inflammatory responses. Additionally, IL1R1 transcription is regulated by NF-κB/JNK/MEK pathways, with p38 MAPK signaling being essential for its expression (28–30). It is noteworthy that although potential differences in gene regulatory networks exist between mouse models and human patients, the fundamental role of IL-1 signaling in propagating inflammatory responses is highly conserved across species. These findings align with the chronic systemic inflammation characteristic of diabetes.

Current evidence indicates that the intervertebral disc functions as an immune-privileged site, with macrophages representing the primary immune cell population involved in disc degeneration. Notably, our scRNA-seq results reveal that beyond monocytes, granulocytes also represent a significant cellular component in diabetes-associated IDD. Analysis of immune cell infiltration for the key diabetes-disc degeneration gene IL1R1 revealed that IL1R1 expression exhibited a significant positive correlation with neutrophils, and a significant negative correlation with activated dendritic cells and activated natural killer cells. Neutrophils, members of the granulocyte family, primarily function to phagocytose and digest invading bacteria and fungi, serving as the first line of defense in the innate immune system. The substantial infiltration of granulocytes revealed by scRNA-seq robustly confirms that the systemic inflammatory response triggered by diabetes is also present within the intervertebral disc. These findings suggest that IL1R1 may play a pivotal bridging role connecting diabetes and intervertebral disc degeneration. We performed qPCR on intervertebral disc tissue from an independent cohort of mice. The results confirmed a significant upregulation of IL1R1 in the diabetic group (p < 0.05), which is highly consistent with our scRNA-seq findings (**Supplementary Figure 1**). However, we did not observe significant morphological differences between the control and diabetic groups under the duration of diabetes induction used in this study according to the H&E staining of disc tissues (**Supplementary Figure 2**). This discrepancy may indicate that diabetes initially drives a molecular and inflammatory pathology within the disc microenvironment, which precedes overt structural degeneration.

The presence of abundant neutrophils within the intervertebral discs of diabetic mice is not surprising. Extensive research has established that neutrophils are prevalent in various chronic diseases, including, besides diabetes, atherosclerosis, non-alcoholic fatty liver disease (NAFLD), and autoimmune diseases (31, 32). Neutrophils, by triggering neutrophil extracellular traps (NETs), can clear senescent vasculature, thereby creating conditions for reparative angiogenesis in ischemic retinas. In diabetic nephropathy, neutrophils can induce glomerular endothelial cell dysfunction and pyroptosis, leading to further kidney damage (33). Moreover, the interaction between neutrophils and platelets is recognized as a key driver of thrombo-inflammation in thrombo-occlusive vascular diseases. Consequently, targeting the mechanisms of platelet–neutrophil interaction, platelet activation/aggregation, and neutrophil recruitment holds promise as a potential therapeutic strategy to mitigate thrombo-inflammation in diabetic patients (34). Our data strongly associate IL1R1 signaling with neutrophil infiltration and NP cell dysfunction under diabetic conditions, the precise mechanistic connections require further investigation. We propose a testable hypothesis: Systemic hyperglycemia and metabolic dysfunction in T2DM lead to the accumulation of AGEs and chronic systemic inflammation. This inflammatory state promotes neutrophil activation and infiltration into the disc environment, likely aided by diabetic microangiopathy and disruption of immune privilege. Once infiltrated, neutrophils serve as a key source of pro-inflammatory cytokines, including IL-1β. The activation of IL1R1 signaling on NP cells—particularly in effector and inflammatory-stress end-stage subpopulations—then triggers downstream cascades via NF-κB and MAPK pathways. This promotes a catabolic phenotype in NP cells, marked by ECM degradation, cellular senescence, and a senescence-associated secretory phenotype (SASP).

The potential link between IL1R1 signaling and cellular senescence is further supported by our GSEA. GSEA of IL1R1 highlighted DNA mismatch repair as the most enriched pathway. Impaired mismatch repair exacerbates DNA damage accumulation, promoting cellular senescence or apoptosis (35, 36). Given the inherently weak repair capacity of terminally differentiated NP cells, senescent or apoptotic NP cells may adopt a SASP, releasing pro-inflammatory factors that amplify degeneration in a vicious cycle (37, 38).

We also constructed an integrated regulatory network centered on IL1R1, incorporating transcription factors (TFs), mRNAs, miRNAs, and lncRNAs. Key regulatory axes included STAT1/IL1R1/hsa-let-7a-3p/LPP-AS2 and STAT6/IL1R1/hsa-miR-125a-3p/LINC. These circuits elucidate the mechanisms sustaining aberrant gene expression in diabetes-associated IDD. Non-coding RNAs (miRNAs, lncRNAs) critically regulate inflammation, extracellular matrix degradation, and senescence/apoptosis—processes that collectively drive IDD (39).

While current clinical management of IDD primarily targets pain relief without addressing core degenerative mechanisms, *in silico* drug screening offers an approach to identify potential therapeutic candidates. Among them, icariin, a natural flavonoid derived from Epimedium, has been suggested by previous pharmacological studies to possess properties relevant to degeneration, such as anti-inflammatory and antioxidant effects (40, 41). Notably, its documented mechanism, antagonizing NF-κB and MAPK-mediated pro-inflammatory cascades, could be conceptually aligned with the IL1R1-driven pathology implicated in our study of diabetes-associated IDD (42). Preliminary molecular docking analysis indicated potential binding modes between icariin and IL1R1, forming a hypothesis for a putative interaction that warrants future experimental validation *in vitro* and *in vivo*.

## Limitations

Our bioinformatics analysis implicates the IL1R1 gene as potentially exerting a profound influence on the occurrence and progression of IDD within the context of diabetes mellitus. However, several limitations warrant acknowledgment. First, the bioinformatics analysis of human transcriptomic data is constrained by the relatively small sample sizes of the publicly available datasets utilized. This limitation may affect the statistical power and generalizability of the identified differentially expressed genes, co-expression modules, and machine learning model. Although we employed rigorous computational methods and cross-validation to generate robust hypotheses, future validation in larger, independent clinical cohorts is essential to confirming the diagnostic utility of the identified biomarkers, particularly IL1R1. Second, it is important to note that the mouse model employed in this study was designed to induce T2DM but not overt IDD through additional mechanical or injury-based means like the needle puncture method. Therefore, we observed significant molecular alterations within the disc cells of diabetic mice. We cannot definitively state that our model recapitulates the full spectrum of structural IDD. This necessitates a cautious interpretation of our findings. The potential causal relationship between IL1R1 expression and the progression of IDD should be viewed as a hypothesis generated from our scRNA-seq data, rather than as an established fact. Future studies utilizing T2DM models combined with controlled disc injury or aging models are essential to conclusively establishing the mechanistic link and causal role of IL1R1 in driving diabetes-accelerated disc degeneration. Third, although bioinformatic analyses suggested the potential therapeutic relevance of icariin, this prediction requires rigorous experimental confirmation. Robust *in vivo* efficacy data in relevant models and evidence from large-scale clinical studies are currently lacking in substantiating its use for IDD. Future work will focus on validating these predictions through *in vitro* and *in vivo* functional studies. Finally, the inherent complexity of diabetes as a metabolic disorder introduces potential confounding factors, such as variations in patient lifestyle, comorbidities, and medication regimens. While our controlled animal model aimed to isolate the effects of hyperglycemia, these human-specific variables could influence gene expression profiles and potentially confound the observed associations in clinical datasets. Future studies incorporating detailed patient stratification and covariate analysis will be essential to translating these findings into the clinical context.

## Conclusion

This study employed integrated bioinformatics approaches to identify three key genes and construct a diagnostic model for diabetes-associated IDD. Notably, IL1R1 emerged as closely associated with this condition and was established as an independent risk factor.

## Data Availability

All public data are reflected in the article. Our own single-cell data have been deposited in the Gene Expression Omnibus (GEO) under the accession number GSE305927.
