# Supplementary figures and images for: Multi-omics analysis and evidence of IL1R1 as a potential biomarker for diabetes-associated intervertebral disc degeneration
Source: Front Immunol. 2025 Oct 15;16:1692185. doi: 10.3389/fimmu.2025.1692185 (PMC12568676; doi:10.3389/fimmu.2025.1692185)

## Relative mRNA expression level of IL-1R1

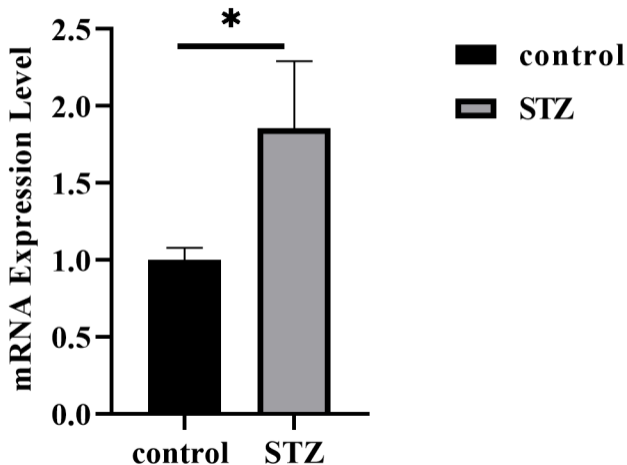

Supplement: Supplementary file 1 [file DataSheet1.pdf]

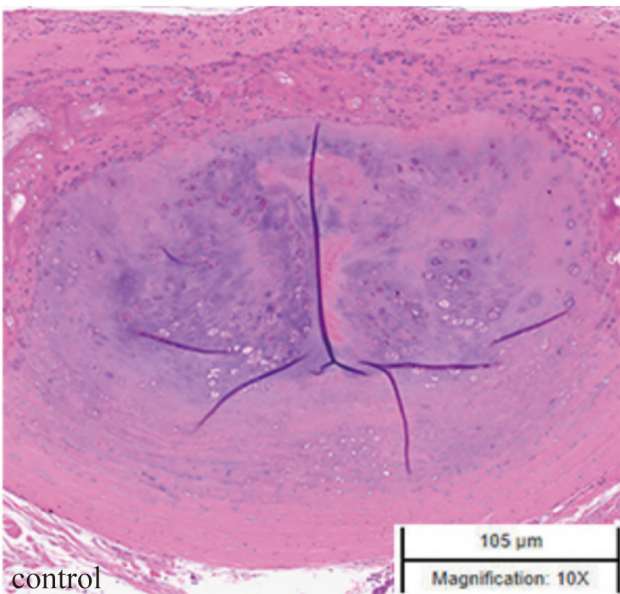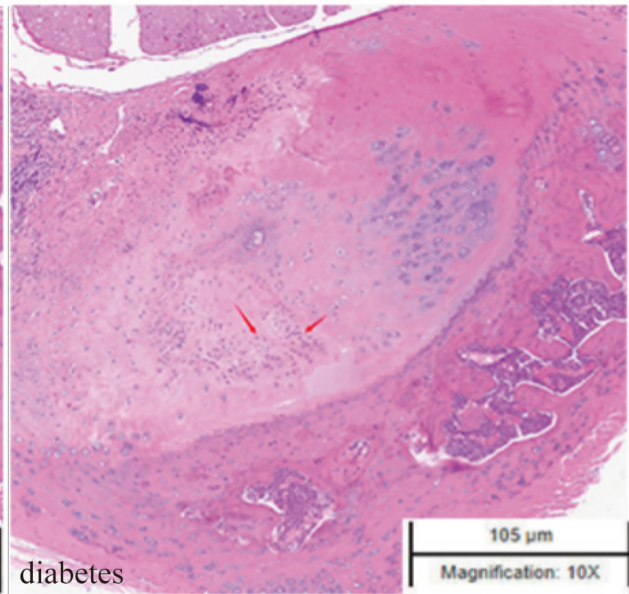

Supplement: Supplementary file 2 [file DataSheet2.pdf]
